# Determinants and perceptions of the utilization of tetanus toxoid immunization among reproductive-age women in Dukem Town, Eastern Ethiopia: a community-based cross-sectional study

**DOI:** 10.1186/s12914-018-0168-0

**Published:** 2018-06-28

**Authors:** Meseret Delesa Anatea, Tesfaye Hambisa Mekonnen, Berihun Assefa Dachew

**Affiliations:** 1 Dukem Health Office, Oromia Regional State, Dukem, Ethiopia; 2 Department of Environmental and Occupational Health and Safety, Institute of Public Health, College of Medicine and Health Sciences, University of Gondar, P.O. Box 196, Gondar, Ethiopia; 3 Department of Epidemiology and Biostatistics, Institute of Public Health, College of Medicine and Health Sciences, University of Gondar, Gondar, Ethiopia

**Keywords:** Tetanus Toxoid Immunization, Reproductive- age women, Cross-sectional, Determinants, Ethiopia

## Abstract

**Background:**

Maternal and neonatal tetanus (MNT) is still the major public health problem in about 25 countries, mainly in Africa and Asia. However, the utilization of intervention strategies, like tetanus toxoid (TT) immunization remains low in these countries. In Ethiopia, only 49% of the pregnant mothers received TT2+ in 2016. This study was designed to evaluate perceptions and factors affecting the utilization of TT immunization among reproductive-age women in Dukem town, Eastern Ethiopia, 2016.

**Methods:**

We conducted a community-based cross-sectional study from May to October 2016. A simple random sampling method was employed to select samples of 422 women. Data were collected using a, pretested semi-structured and a face-to-face interviewer-administered questionnaire. We entered data in to Epi Info version 7 and analyzed them by SPSS version 20 software. Odds ratios and a 95% CI at 0 < 0.05 p-value were calculated to ascertain the significance of associations.

**Results:**

Response rate was 98.6% (*N* = 416). Mean age with standard deviation was 29.25± 5.11 years, and average family size was 4.19. Our study showed the utilization of TT immunization was 39.2% (*N* = 163). Of the participants, 33.9% (*N* = 141) had never been vaccinated. ANC follow up service [AOR: 2.56, 95% CI: (1.18, 5.49)], distance from health facilities [AOR: 2.27, 95% CI: (1.27, 4.09)], knowing vaccination date [AOR: 1.98, 95% CI: (1.23, 3.18)], having a TV set in the house [AOR: 1.80, 95% CI: (1.11, 2.917)], maternal education [AOR: 1.41, 95% CI: (1.84, 2.30), and place of delivery [AOR: 1.19, 95% CI: (1.00, 1.43)] were factors significantly associated with the utilization of TT immunization.

**Conclusions:**

This study indicated the utilization of TT immunization was low. ANC service follow up, distance from health facilities, knowing vaccination date, having a TV in the house, mothers’ educational status, and place of delivery were significant predictors. Our study suggests that policymakers and other stakeholders should consider the need for increasing access to maternal education, like basic adult education, ANC follow up services, providing accessible health facilities, improving varieties of communication media, promoting female occupational status, and providing appropriate vaccination cards.

## Background

Maternal and neonatal tetanus (MNT) is still the major public health problem, in about 25 countries, mainly in Africa and Asia [1–3]. Maternal and neonatal tetanus is a condition which occurs under poor hygienic practices during delivery performances [4]. Tetanus which occurs during pregnancy or within 6 weeks of the end of pregnancy is called maternal tetanus (MT) and neonatal tetanus (NT) is tetanus that occurs in the first 28 days of life [5]. NT is an acute disease caused by a tetanus toxin produced by bacteria called Clostridium Tetani. The spores enter the body through an unhealed umbilical cord [6].

Despite the initiatives of the World Health Organization (WHO) for tetanus elimination, it still continues to cause a significant maternal and neonatal mortality. Annually, it claims about 180,000 lives worldwide [4]. In the poorest parts of the world, neonatal tetanus (NT) is responsible for 14% of the neonatal deaths, while maternal tetanus (MT) is responsible at least for 5% of maternal deaths [7]. An estimated 15,000–30, 000 women have been dying every year from tetanus contracted during or shortly after pregnancy [8].The risk for tetanus in women and neonates is especially high in developing countries. This is because in such countries,women are not well protected through full tetanus immunization; persistent unsafe delivery practices or limited accessibility prevail; and unhygienic cord care practices continue [9].In 1999, WHO estimated that about 17,875 neonatal tetanus cases and 13,406 NT deaths took place in Ethiopia making the country a contributor of 4.6% of the global NT deaths [10].

Both maternal and neonatal tetanus are preventable health problems. Proper implementations of maternal immunization programs, like the TT immunization is one of the basic intervention strategies used to alleviate these situations. Tetanus toxoid immunizations are injections given during pregnancy for the prevention of NT. A pregnant woman should receive at least two doses of TT injections during each pregnancy to realize full tetanus protection. Five doses are considered to provide lifetime protection irrespective of the recommended intervals [11]. Studies proved that immunization of pregnant women or women of childbearing age with at least two doses of TT could have the potential to decrease mortality from NT [6, 12]. Moreover, clean delivery practices and appropriate tetanus surveillance are the other basic approaches for MNT prevention [5, 9, 12].

In many parts of low-income countries, however, despite the efforts made to improve maternal and neonatal protection through TT immunization programs, valid doses of TT (two and more) immunization coverage still remains low [3]. For instance, about 27% of the participant women did not receive any TT vaccination during their most recent pregnancies in India [13]. A report from Pakistan demonstrated that only 55.6% of the participants received the complete TT vaccination [14].

Similarly, the majority of the Sub-Saharan African countries could hardly reach the TT immunization target set to be covered. Because of this, they have not yet fully achieved the complete elimination of MNT. Accordingly, a study from Nigeria showed that only 40.8% of TT2 coverage was achieved while 44.4% of mothers did not receive any TT vaccination [15]. Ethiopia is one of the countries which have not fully achieved the complete elimination of MNT [5, 16] due to the low TT coverage achieved. According to Ethiopian Demographic and Health Survey (EDHS) reports of 2016, only 49% of women received sufficient doses of TT (TT2+) [17]. It could be concluded that most African countries could not reach the WHO Global Immunization target of at least 90% national vaccination coverage and at least 80% vaccination coverage in every district [18].

Evidences show that a range of determinant factors affect the utilization of TT immunization. For instance, women’s education and their wealth index can impose variations in immunization coverage [17, 19, 20]. Mothers’ immunization status also differs from urban to rural as well as from one part of the country to the other [21, 22]. Furthermore, studies indicate that maternal age, marital and occupational status, distance from health facilities, number of visits to healthcare facilities, and the number of children in the house can also considerably determine TT immunization usage [22–24].

Surprisingly, we lack evidences demonstrating the number of factors influencing the utilization of TT immunization and mothers’ perceptions of the immunization in Dukem town, East Showa Zone, Ethiopia. Thus, this study intended to investigate the level and ranges of factors affecting the utilization of TT immunization among reproductive age women in Dukem town, Eastern Ethiopia. The finding would probably help us to generate supplementary evidences that strengthen and accelerate current efforts to eliminate MNT.

## Methods

### Aims

This study was intended to investigate the status and factors determining the utilization of TT immunization among reproductive age women in Dukem town, Eastern Ethiopia.

### Study design, setting, and period

A cross-sectional study design was employed to assess the level and factors affecting the utilization of TT immunization. We collected data based on the number of reproductive -age women currently living in Dukem town from May to October 2016.

### Study area

Dukem town, which is located 35 km to the East of Addis Ababa, the capital of Ethiopia, was our study area. We purposively selected the town and its surroundings. In its four kebeles, the town had a projected total population of 114, 207 according to the 2007 population and housing census of the Central Statistical Agency (CSA) of Ethiopia [25]. Child bearing age women (CBAW) were estimated to be 25,234 (22.2%) of the total population, and approximately 3957(3.47%) of them became pregnant annually. The health facilities of the town during the study included a public health center, a maternal and child health (MCH) clinic, four public health posts and eight medium private clinics.

### Source population

The source population was all child bearing age women who were living in Dukem town at the moment.

### Inclusion and exclusion criteria

#### Inclusion criteria

All women in the reproductive age group who had lived in the study area for at least two years were included.

#### Exclusion criteria

We excluded all reproductive age women who had never given births in the past 2 years.

### Sample size and Sampling procedures

We employed the simple random sampling method to select eligible participants. Participant mothers were proportionally selected based on the number of resident mothers in each of the 4 kebeles. Single population proportion was used to calculate the required sample size. A 50% assumption of prevalence and an absolute precision of 5% were considered. We also assumed of a 95% confidence level to obtain adequate power for analysis. After including an additional grant of 10% for no response, 422 women were included.

### Operational definitions

**♣ Child Bearing Age Women (CBAW) -** Any women aged 15 to 49 years irrespective of fertility status.

**♣ Vaccinated by history**: Mothers’ self-report of TT vaccination doses received without any documented evidences.

**♣ Vaccinated by card only**: A documented evidence of TT doses on immunization cards only.

**♣ Vaccinated by card plus history**: Both documented and mothers’ self-reported TT doses received.

**♣ Valid TT doses**: Mothers who received at least 2 doses of TT (TT2) in the recommended intervals

### Data collection tools and techniques

We collected data by using a semi-structured interviewer administered questionnaire. The questionnaire was adopted from the Ethiopian demographic and health survey and other related literature [10, 19, 20, 24–26]. We employed a face-to-face interviewer administered data collection technique. We included age, mothers’ marital status, mother and husband’s educations, mothers’ ethnic group, religion, radio and TV in the house to gather information on the socio-demographic characteristics of the participants. There also were questions about mothers’ future birth intentions, ANC service follow ups, parity of birth, permission from husbands to go to health facilities, and knowing the next date of TT vaccination dose to evaluate the determinants of TT vaccination among participants.

Questions like the accessibility of vaccination site, quality of services provided, behavior of health care providers, time for travel to health facilities, privacy issues during services, and husbands’ encouragement to visit HF were also incorporated to assess mothers’ perceptions toward the utilization of TT vaccinations. We also asked some questions concerning mother’s knowledge of the purpose of TT immunizations.

### Data quality control

We emphasized quality in the data collection tools. The questionnaire was first designed in English and translated into the local language ‘Afaan Oromoo’ and back to English by language experts to verify its consistency. We also recruited 7 data collectors and 5 supervisors (health extension workers) who had experience and skills in the task. They were trained and oriented for 2 days before the actual survey. The training focused on the purposes of the study, clarity of tools, techniques of interview, confidentiality of information, informed consent, and the roles & responsibilities of data collectors and supervisors. During the data collection process, the principal investigator supervised both groups. Moreover, we conducted a pretest study prior to the actual process to test the validity and consistency of the data collection instrument by using 10% of the sample in a neighboring town, Gelan. Based on the pretest analysis, we modified some misinterpretations, minimized the number of interview questions, and made corrections to some other objections.

### Data management and analysis

The collected data were checked manually for clarity and completeness. We coded data, labeled, verified, categorized, and entered into EpiInfo version 7. We employed SPSS version 20 to analyze the data. Descriptive statistics, like frequencies, percentages, means and standard deviations were computed. Using a binary logistic regression analysis, we fitted each predictor variable in to a bivariate logistic regression model separately to explore associations with the dependent variable (TT immunization status). Significant predictors at p-value < 0.2 in a bivariate analysis were exported to the multivariable logistic regression model to control possible effects of confounders. Variables were dropped in to the multivariate logistic regression model with a backward variable selection method. We proved goodness of fit model by Hosmer and Lemeshow and the assumption was satisfied (*p*-value > 0.05). Odds ratios (OR) with 95% confidence intervals (CI) were applied to ascertain the significance of association.

## Results

### Socio-economic characteristics

Out of the total 422 sampled mothers, 416 (98.6%) fully responded to the interview questionnaire and included in the analysis. The mean age was 29.25 years and the standard deviation + 5.11. The majority, 414 (99.5%), of the respondents were in the age group of 19–43 years. Average family size was 4.19. Seventy-eight (18.8%) of the respondents never attended any formal school. The majority of the participants, 295 (70.9%) belonged to the Oromo ethnic group (Table 1).

**Table 1:** Socio-demographic characteristics of the participants in Dukem town, Ethiopia, 2016

Variables (*N* = 416)	Frequency	Percentage (%)
Religion		
Orthodox	263	63.2
Catholic	20	4.8
Protestant	105	25.2
Muslim	28	6.7
Maternal education		
Never attended	78	18.8
Only read & write	92	22.1
primary school	112	26.9
Secondary and above	134	32.2
Husband education		
Never attended	60	14.4
Only read & write	68	16.3
Primary school	95	22.8
Secondary and above	193	46.4
Maternal ethnic group		
Oromo	295	70.9
Amhara	90	21.6
Guraghe	22	5.2
Tigrie	9	2.3
Mothers marital status		
Married	328	78.8
Divorced	49	11.8
Widowed	15	3.7
Never married	12	2.9
Separated	12	2.8
Monthly income		
≤500ETB	28	6.7
501-1000ETB	93	22.3
> 1000ETB	295	71.0
Radio in the house		
Yes	249	59.9
No	167	40.1
TV in the house		
Yes	261	62.7
No	155	37.3

Keys:-*EBT* Ethiopian Birr; *N* number; *TV* Television

### Determinants of TT immunization utilization

Two hundred fifty one (60.3%) of the participants gave birth to 2–4 children in their life. The majority, 218 (52.4%), of the mothers did not have future intention of giving birth. Two hundred seventy-five (66.1%) mothers visited health institutions less than 4 times for ANC services in their last pregnancy. Of the participants, 243 (58.4%), reported that the evidence of TT immunization they utilized was history (Table 2).

**Table 2:** Determinants of the utilization of TT immunization, Dukem town, Ethiopia, 2016

Variables (*N* = 416)	Numbers	Percentages (%)
Parity of Birth		
1	142	34.2
2–4	251	60.3
≥ 5	23	5.5
Future intention of birth		
wants	142	34.1
Don’t want	218	52.4
Not decided	56	13.5
Permission of husband to go to HF		
No restrictions	204	49.0
Restricted some times	143	34.4
Restricted at all	69	16.6
Follow up of ANC services		
Yes	363	87.2
No	53	12.8
Number of ANC services Visited		
Once	71	17.1
twice	92	22.1
three times	112	26.9
Four or more times	141	33.9
TT vaccination evidences		
By history	243	58.4
By card	173	41.6
Vaccination status		
Not vaccinated	141	33.9
TT1	112	26.9
TT2+	163	39.2
Knowing the next TT vaccination dates		
Yes	293	70.4
No	123	29.6
Place of deliver		
Health institution	276	66.8
At home	138	33.2

Keys: -*ANC* Antenatal care; *HF* Health facilities; *TT* Tetanus toxoid

### Valid TT doses utilization status

**Fig. 1:**
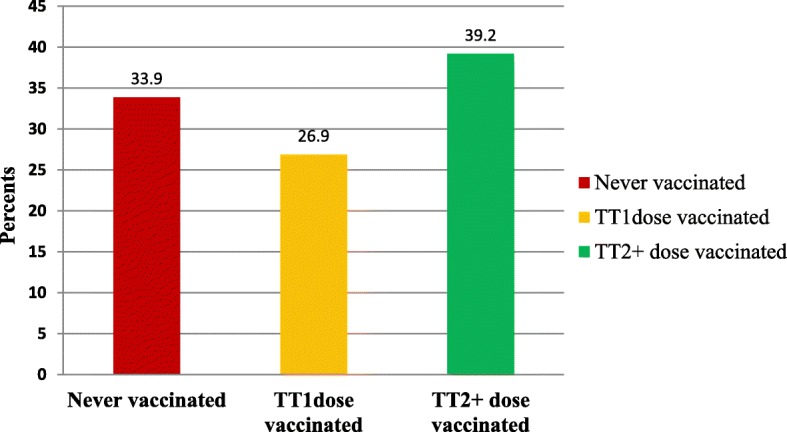
Valid TT immunization utilization status in Dukem Town, Western Ethiopia, 2016

In this study, a significant proportion of the mothers, 33.9% (*N* = 141) [95% CI: (29.6, 38.2)] reported they were never vaccinated with any doses of TT drug and had no documentation (card). Out of the interviewed mothers, only 39.2% (*N* = 163) were vaccinated the valid (TT2+) doses, and one hundred twelve (26.9%) [95% CI: 22.8, 31.0] were vaccinated by the TT1 vaccination dose (Fig. 1).

### Mothers’ perceptions towards utilization of TT vaccination

**Fig. 2:**
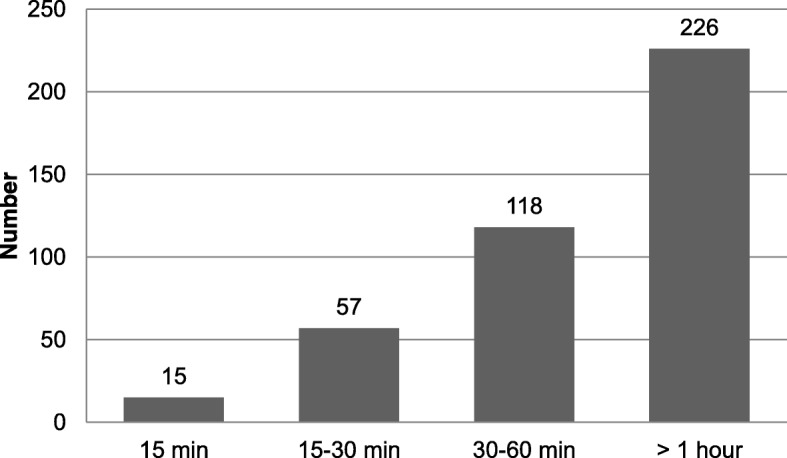
Mothers’ waiting time during TT services at health facilities, Dukem Town, Western Ethiopia, 2016

We also interviewed the participants about their perceptions of TT vaccination utilization. A high proportion, 87.5% (*N* = 364) of the participants indicated that there were accessible vaccination sites closest to their dwellings. One hundred ninety-eight (47.6%) of the mothers ranked the services provided as “good”. More than half, 54.3% (*N* = 226) of the participant mothers pointed out waiting time for services at facilities was> 1 h (Fig. 2 & Table 3).

**Table 3:** Participants’ perceptions towards utilization of TT immunization, 2016 (*N* = 416)

Variables	Frequency	Valid Percent
Accessibility of vaccination site		
Yes	364	87.5
No	52	12.5
Where the health workers respectful		
Yes	250	60.0
No	158	37.9
Don’t know	8	2.1
Lack of privacy is a problem		
Yes	84	20.2
No	270	64.9
Don’t know	62	14.9
Quality of service given		
Good	198	47.6
Satisfactory	161	38.7
Poor	48	11.5
Don’t know	9	2.2
Behaviors of health workers		
Very Good	70	16.8
Fair	287	69.0
Bad	59	14.2
Cancelation of vaccine schedule		
Yes	179	42.2
No	237	57.8
Reason of schedule cancelation		
For my health problem	25	6.0
Lack of information	115	27.6
Lack of drug	39	9.4
Those who are not canceled and started	237	57.0
Complains TT drug after vaccination		
Health Effects	98	23.6
Others effects	18	4.4
No Effects and not vaccinated	300	72.0
Time to travel to HF		
15 min	37	8.9
15–30 min	146	35.1
30–60 min	161	38.7
>one hour	72	17.3
Husband encourages to visit HF		
Yes	280	67.3
No	136	32.7

Keys: - *TT* Tetanus toxoid; *N* number; *min* minutes; *HF* health facilities

### Mothers’ perceived purposes of TT immunization

**Fig. 3:**
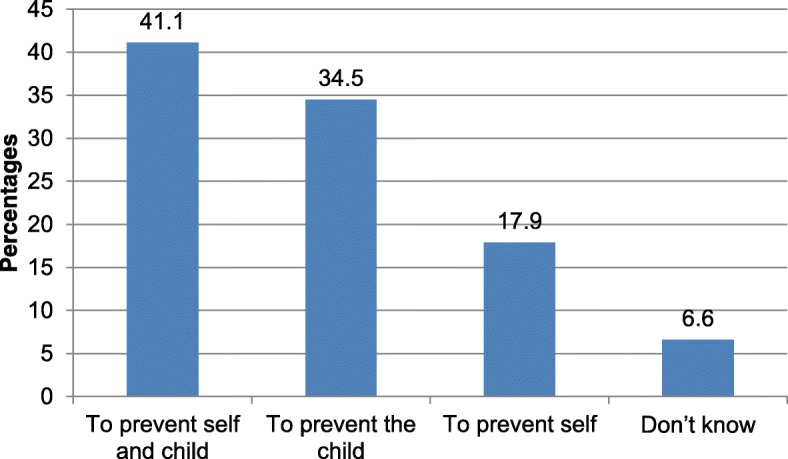
Mothers’ perceived purposes of TT immunization, Dukem Town, Western Ethiopia, 2016

Figure 3 below shows the knowledge of mothers about the purposes of TT immunizations. Of the mothers who were vaccinated valid TT doses, 40.9% (N = 67) stated the purpose of TT immunization was to prevent mothers and children from getting tetanus disease, while 34.5% (*N* = 56) said the purpose was to prevent children alone (Fig. 3).

### Factors affecting utilization of TT immunization

Bivariate logistic regression analysis demonstrated that explanatory variables, like maternal education, occupational status of mothers, monthly income, TV set in the house, parity of birth, ANC service follow up, knowing TT vaccination dates, distance from health facilities, and place of birth were considerably related to maternal TT immunization status.

After controlling possible sources of confounders in the multivariable logistic regression model, mothers’ educational level, TV set in the house, occupational status of mothers, ANC service follow up, knowing TT vaccination date, distance from health facilities, and place of birth indicated a significant association with the utilization of TT vaccination. Accordingly, literate mothers were 1.41 times more likely to be immunized than illiterate mothers [AOR: 1.41, 95% CI: (1.18, 2.30)]. Participants who used TV in their house showed 1.8 times higher chance for TT immunization usage [AOR: 1.80, 95% CI: (1.11, 2.92)]. The multivariable logistic regression model also showed that mothers utilizing ANC service follow ups were 2.56 times more likely to utilize TT immunization [AOR: 2.56, 95% CI: (1.18, 5.49)] than those who did not follow ANC services. Moreover, mothers who were far from health facilities by < 30 min were 2.27 times more likely to be immunized compared to those who were far by ≥ 30 min [AOR: 2.27, 95% CI: (1.27, 4.09)]. The model also explained that respondents who knew TT vaccination dates had 1.98 times higher possible utilization of TT immunization [AOR: 1.98, 95% CI: (1.23, 3.18)] compared to who did not know vaccination dates (Table 4).

**Table 4:** Factors affecting the utilization of TT immunization, Dukem town, Ethiopia, 2016

Variables (*N* = 416)	TT Immunization Status		COR (95% CI)	AOR (95% CI)	*p-value*
	≤TT1	TT2+			
	*n* = 253	*n* = 163			
Maternal education					
Illiterate	146(25.5%)	50(74.5%)	3.03(2.03,4.47)	1.41(1.84, 2.30)	0.003+
Literate	107(48.6%)	113(51.4%)	1	1	
Maternal occupation					
Employed	153(67.4%)	74(32.6%)	1.84(1.23,2.74)	1.27(1.08,1.49)	0.011+
Non-employed	100(52.9%)	89(47.1%)	1	1	
Monthly income					
< 1000 ETB	88(67.8%)	40(31.2%)	1.62(1.04,2.52)	1.2(0.02,1.39)	0.03^a^
> 1000 ETB	165(57.5%)	122(42.5%)	1	1	
TV in the house					
Yes	186(65.7%)	97(34.3%)	1.64(1.09,2.47)	1.80(1.11,2.92)	0.002+
No	77(53.8%)	66(46.2%)		1	
Parity of birth					
< 2	174(63.5%)	100(36.5%	1.38(1.02,2.26)	1.14(0.96,1.36)	0.03^a^
> 2	79(55.6%)	63(44.4%)	1	1	
ANC service follow up					
Yes	97(72.9%)	36(27.1)	2.2(1.40,3.43)	2.56(1.18,5.49)	0.002+
No	156(54.6%0	127(45.4%)	1	1	
Knowing vaccination date					
Yes	93(85.3%)	16(14.7%)	5.3(3.00,9.5)	1.98(1.23,3.18)	0.005+
No	160(52.1%)	147(47.9%)	1	1	
Distance from HF					
< 30 min	122(66.7%)	61(33.3%)	1.56(1.04,2.32)	2.27(1.27, 4.09)	0.001+
≥ 30 min	131(56.2%)	102(43.8%)	1	1	
Place of delivery					
Health facility	180(64.3%)	100(35.7%)	1.60(1.02,2.36)	1.19(1.00,1.43)	0.004+
Home	73(52.9%)	63(47.1%)	1	1	

Keys: - ^a^ **=** significant in a bivariate analysis; + = significant in a multivariable analysis; *AOR* Adjusted odds ratios; *CI* Confidence interval; *COR* Crude odds ratios; *ETB* Ethiopian Birr; *HF* Health facilities; *TT* Tetanus toxoid; *TV* Television

## Discussion

This study employed a community-based quantitative cross-sectional study to explore factors affecting the utilization of TT immunization and perceptions of the service among reproductive age women in Dukem town, Eastern Ethiopia. The study also aimed to quantify the level of the utilization of TT immunization in the area. We found the prevalence of valid TT dose (≥TT2+) utilization was 39.12% [95% CI: (32.8, 41.0)]. The study also revealed that only 41.1% of the participants used TT vaccination by card. In this study, a notable proportion (33.9%) of the participants were never vaccinated any dose of TT.

Our finding indicated TT immunization utilization status was relatively lower compared to the national 49% reported by the Ethiopian Demographic and Health Survey (EDHS) of 2016 [17] and a prevalence of 60.8% from other parts of the country [19]. This disparity might be due to lack of uniform performance commitment to implement TT vaccination services in all regions of the country. This is in line with other study reports in that immunization coverage varies among communities of the same territory [11].

Our finding was also lower than the TT vaccination status reported by studies in Pakistan [14] and Bangladesh [11], which was 55.6% in both countries, and prevalence of 61.4% TT utilization reported from Kenya [27] and 68% from India [28]. This could be due to differences in cultural and socio-economic factors such as the level of knowledge and information about vaccination, healthcare delivery systems, like the availability of health centers for vaccination, the prevalence of vaccine-preventable diseases, methodological differences in measuring immunization status, and the existing political environment [29].

Our finding indicated the level of maternal education, maternal occupational status, ANC service follow up, distance from health facilities, media exposure, knowing vaccination date, and places of delivery considerably predicted the utilization of TT immunization.

The result of the multivariate logistic regression model explained that mothers’ educational status was significantly associated with the utilization of TT immunization. Hence, literate mothers were significantly more likely to be immunized than illiterate mothers. This result was consistent with other study reports [11, 15, 19, 28, 30–32]. The possible reason might be due to differences in knowledge between the two groups about TT vaccination services. Educated mothers are often more confident decision makers regarding their own care-seeking behaviors. Because of this, educated women are often granted more status in most social interactions. In addition, care providers treat them more respectfully since they can easily communicate with them. Other studies also stated similar explanations [20, 28, 33].

The 2016 EDHS report [17] also found a similar result. The survey showed that a large number of the women (83%) whose last live births were protected against NT were more educated (over secondary school) compared to non-educated women only 41% of whom had protected live births. Therefore, maternal education is a vital tool through which we can realize the utilization of TT immunization coverage. Ensuring access to maternal education such as the expansion of basic adult education and other forms of informal educational coverage for women should go hand in hand with a successful implementation of TT immunization.

In this study, maternal ANC service follow up markedly increased the likely hood of the utilization of TT immunization. Women who regularly attended ANC service follow ups had a higher probability of TT vaccination utilization. This was supported by other findings [14, 23, 27, 31, 34, 35]. The probable explanation might be that frequent contact with healthcare providers itself upholds and boosts awareness about the benefits of utilizing complete TT vaccination. The other possible reason might be that, in Ethiopia, TT immunization is one of the ANC service packages. On the other hand, the national EDHS report of 2016 showed that 62% of women who gave birth in the last five years preceding the survey received ANC services. It could be concluded that there were a substantial number of missed opportunities for TT immunization within the same period as only 49% immunization that was too far from the ANC service was achieved. Care providers in different health facilities in the country should fill this gap by taking a better advantage of women’s ANC attendance opportunities throughout the ANC service provision procedures. Moreover, together with the recent advent of the national health policy, which focuses on accessible health service coverage, the newly gained experiences of health care services which may result in missed opportunities for vaccination have to be managed with due care. Moreover, the information provided during the services could also help to shape women’s attitude towards subsequent health services. The existence of such missed opportunities is common in other countries too [36].

Our analysis detected distance from health facilities was an important predictor of TT immunization status. In concordance with other studies [14, 37], women whose travel required only < 30 min to reach the nearest health centers had a higher chance for the utilization of TT immunization. This could be because of costs associated with distant vaccination centers in terms of time and transportation expenses. Women are most often busy at home because they are responsible for multiple responsibilities, such as caring for children and older people as well as other house hold activities. Therefore, they often hesitate to visit vaccination centers, which are relatively far from their homes. Moreover, since TT vaccination requires repeated visits to health facilities for complete immunization, such recurrent visits might be tiring for women and their children if vaccination centers are relatively far away. Ensuring accessible health facility coverage in every part of the region could lessen such problems.

The odds of TT immunization status were more likely to increase among employed mothers compared to non-employed ones. A number of studies demonstrated that TT immunization utilization depended on mothers’ occupational status [23, 27, 38, 39]. Our finding also corroborated these reports. This might be so because employed women no doubt have more access to better information and communication about health services in general and TT immunization in particular than unemployed women. Moreover, work place itself is an ideal place to promote and engage women in different health related issues, thereby enhancing women’s utilization of health care services.

Place of delivery was another determinant factor for the utilization of TT immunization. In this study, we found that mothers who gave birth at health facilities were highly likely to use TT immunization compared to those who gave birth at home. Other studies also verified this association [24, 29, 30, 35, 38]. The possible reason might be because of the opportunity for health education and advice when women deliver at health facilities. It might also be due to opportunities to provide mothers with at least the first dose of TT just immediately after delivery procedures.

Similar to the other studies [38], our analysis confirmed that knowing vaccination dates increased the odds of TT immunization utilization. A possible suggestion could be the number of contacts between care providers and client mothers. Frequent contacts with health workers could remind about the correct immunization schedules. It is important to use notification strategies such as notification through local authorities and other community leaders for vaccination dates.

Women’s media exposure, like having TV sets in houses significantly determined the status of TT immunization. In line with other studies [20, 24, 29, 38], our finding showed that women who had TV sets in their houses were more likely to indicate the utilization of TT immunization. A possible explanation might be that TV is the major information dissemination media for health service utilization. It is advisable to use alternative communication media, like the radio, school community, posters, and various community meetings.

Although we found out reliable information that can add to existing literature, some limitations could be distinguished in this study. First, the data collected were based on events that happened within the last two years preceding the study. Therefore, the problem of recall bias and under reporting might be suspected. To decrease such limitations, we focused on the most recent births and utilized vaccination cards and other records of the health facilities. Second, since the data collection technique was a face-to-face interview, there might be a social desirability bias. However, to address this issue, we carefully focused on interview techniques when we trained data collectors and supervisors.

## Conclusions

This study indicated the utilization of valid TT dose immunization was low in the area. Maternal education, media exposure, ANC follow up services, knowing vaccination date, distance from health facilities, and place of delivery were significant predictors of the utilization of TT immunization. The study suggests that policy makers and other stakeholders should consider the need to ensure access to maternal education, like basic adult education. Moreover, it is advisable to promote ANC follow up services and to provide access to health facilities, varieties of communication media coverage, and appropriate vaccination cards accessible to remind about remaining TT doses.

## Abbreviations

ANC: Antenatal care
AOR: Adjusted odds ratios
CI: Confidence interval
COR: Crude odds ratios
CSA: Central statistical agency
EDHS: Ethiopian demographic and health survey
HF: Health facility
MCH: Maternal and child health
MN: Maternal tetanus
MNT: Maternal and neonatal tetanus
MPH: Masters of public health
MSC: Masters of science
NT: Neonatal tetanus
SPSS: Statistical package for social science
TT: Tetanus toxoid
WHO: World Health Organization
